# A nomogram based on CT intratumoral and peritumoral radiomics features preoperatively predicts poorly differentiated invasive pulmonary adenocarcinoma manifesting as subsolid or solid lesions: a double-center study

**DOI:** 10.3389/fonc.2024.1289555

**Published:** 2024-01-19

**Authors:** Zebin Yang, Hao Dong, Chunlong Fu, Zening Zhang, Yao Hong, Kangfei Shan, Chijun Ma, Xiaolu Chen, Jieping Xu, Zhenzhu Pang, Min Hou, Xiaowei Zhang, Weihua Zhu, Linjiang Liu, Weihua Li, Jihong Sun, Fenhua Zhao

**Affiliations:** ^1^ Department of Radiology, Affiliated Dongyang Hospital of Wenzhou Medical University, Dongyang, China; ^2^ Department of Radiology, Affiliated Xiaoshan Hospital of Wenzhou Medical University, Hangzhou, China; ^3^ Department of Radiology, Sir Run Run Shaw Hospital, Zhejiang University School of Medicine, Hangzhou, China; ^4^ Department of Radiology, Fourth Affiliated Hospital, College of Medicine, Zhejiang University, Yiwu, China; ^5^ Department of Pathology, Affiliated Dongyang Hospital of Wenzhou Medical University, Dongyang, China; ^6^ Medical Imaging Department, Shenzhen Second People’s Hospital/the First Affiliated Hospital of Shenzhen University Health Science Center, Shenzhen, China; ^7^ Cancer Center, Zhejiang University, Hangzhou, China

**Keywords:** pulmonary adenocarcinoma, computer tomography imaging, radiomics, peritumoral, nomogram

## Abstract

**Background:**

The novel International Association for the Study of Lung Cancer (IASLC) grading system suggests that poorly differentiated invasive pulmonary adenocarcinoma (IPA) has a worse prognosis. Therefore, prediction of poorly differentiated IPA before treatment can provide an essential reference for therapeutic modality and personalized follow-up strategy. This study intended to train a nomogram based on CT intratumoral and peritumoral radiomics features combined with clinical semantic features, which predicted poorly differentiated IPA and was tested in independent data cohorts regarding models’ generalization ability.

**Methods:**

We retrospectively recruited 480 patients with IPA appearing as subsolid or solid lesions, confirmed by surgical pathology from two medical centers and collected their CT images and clinical information. Patients from the first center (n =363) were randomly assigned to the development cohort (n = 254) and internal testing cohort (n = 109) in a 7:3 ratio; patients (n = 117) from the second center served as the external testing cohort. Feature selection was performed by univariate analysis, multivariate analysis, Spearman correlation analysis, minimum redundancy maximum relevance, and least absolute shrinkage and selection operator. The area under the receiver operating characteristic curve (AUC) was calculated to evaluate the model performance.

**Results:**

The AUCs of the combined model based on intratumoral and peritumoral radiomics signatures in internal testing cohort and external testing cohort were 0.906 and 0.886, respectively. The AUCs of the nomogram that integrated clinical semantic features and combined radiomics signatures in internal testing cohort and external testing cohort were 0.921 and 0.887, respectively. The Delong test showed that the AUCs of the nomogram were significantly higher than that of the clinical semantic model in both the internal testing cohort(0.921 vs 0.789, p< 0.05) and external testing cohort(0.887 vs 0.829, p< 0.05).

**Conclusion:**

The nomogram based on CT intratumoral and peritumoral radiomics signatures with clinical semantic features has the potential to predict poorly differentiated IPA manifesting as subsolid or solid lesions preoperatively.

## 1 Introduction

Lung cancer is the prominent reason for cancer-related death globally (1). Non-small cell lung cancer (NSCLC) occupies approximately 80%-85% of lung cancer cases, the most common type of which is adenocarcinoma (2). Lung adenocarcinoma comprises five frequent histological subtypes, which were reported to indicate patients’ prognoses (3). In 2015, the WHO divided patients into 3 prognostic groups based on the 5 pathological subtypes: low-grade pattern (lepidic predominance), intermediate-grade pattern (acinar or papillary predominance), and high-grade pattern (solid or micropapillary predominance) (4). However, many scholars found some limitations to this classification method. First, minor subtypes’ prognostic impact has not been considered. For instance, cases with solid or micropapillary components (even not dominant) usually have poor prognoses (5). Moreover, a new complex glandular pattern has been identified but not included in the grouping, which correlates to high mitotic rates, tumor necrosis, and lymphatic invasion (6). It is agreed that this novel pattern represents a similar prognosi (7–9) to adenocarcinoma with solid or micropapillary predominance.

In 2020, the International Association for the Study of Lung Cancer (IASLC) introduced a newly modified grading system (10) combining dominant subtype and high-grade components. Tumors with ≥20% high-grade patterns (solid, micropapillary, or complex glandular patterns) are delineated as poorly differentiated tumors (PDT). The novel grading system was adopted by the WHO thoracic tumor classification (5th edition) in 2021 (11). Several large-scale cohort studies have corroborated that the new classification system bears substantial prognostic predictive power (12–14). Besides, a recent study demonstrated that the 3-year recurrence-free survival (RFS) rate of PDT is only 65.5%, while the 3-year RFS of non-poorly differentiated tumor (n-PDT) is 88.3%-100% (12). Stage IA NSCLC can be treated with segmentectomy (13), which helps minimize surgical trauma and preserve more lung function. Yet, Xu et al. (14) reported that PDT is a practical predictive index of mediastinal lymph node metastasis in clinical stage I invasive pulmonary adenocarcinoma (IPA). Patients with PDT may need more thorough radical surgery and mediastinal lymph node dissection in the early stage. Although IPA can be determined through needle biopsy or intraoperative frozen sections, it is challenging to diagnose PDT, which requires complete pathological tissue (10). This is crucial for thoracic surgeons, as it may influence the choice of surgical approach. Therefore, there is an urgent need to establish an accurate and generalizable model for preoperative prediction of PDT to help IPA patients receive the most appropriate treatment.

Radiomics is a non-invasive and reproducible approach that quantifies copious objective high-dimensional quantitative data to demonstrate tumor heterogeneity (15). PDT has a poor prognosis and is predisposed to relapse and metastasis, probably attributed to the peritumoral stroma, inflammation level, lymphatic infiltration, vascular infiltration, etc. (16–18). Nevertheless, the spatial heterogeneity above is tough to be observed in CT images. Radiomics seems able to decipher the peritumoral microenvironment. Some studies have corroborated that combining intralesional and perilesional radiomics features can better discriminate between benign and malignant pulmonary lesions (19) and predict tumor invasiveness (20). Moreover, previous research has shown that radiomics can differentiate the histological subtypes of lung adenocarcinoma (21). Recent studies (22, 23) reported that radiomics could also forecast poorly differentiated IPA in the novel IASLC classification. However, they neglected essential peritumoral prediction or lacked external testing with independent datasets. No radiomics research has incorporated peritumoral data to predict PDT under the novel IASLC classification with independent dataset testing. IPA radiologically manifesting as pure ground-glass opacity is a group of tumors with a very low risk of metastasis and recurrence, resulting in an excellent prognosis (24–26). We aim to investigate the nomogram’s potential to predict poorly differentiated IPA manifesting as subsolid or solid lesions based on intratumoral and peritumoral radiomics features combining clinical semantic features.

## 2 Material and methods

### 2.1 Patient selection

**Figure 1:**
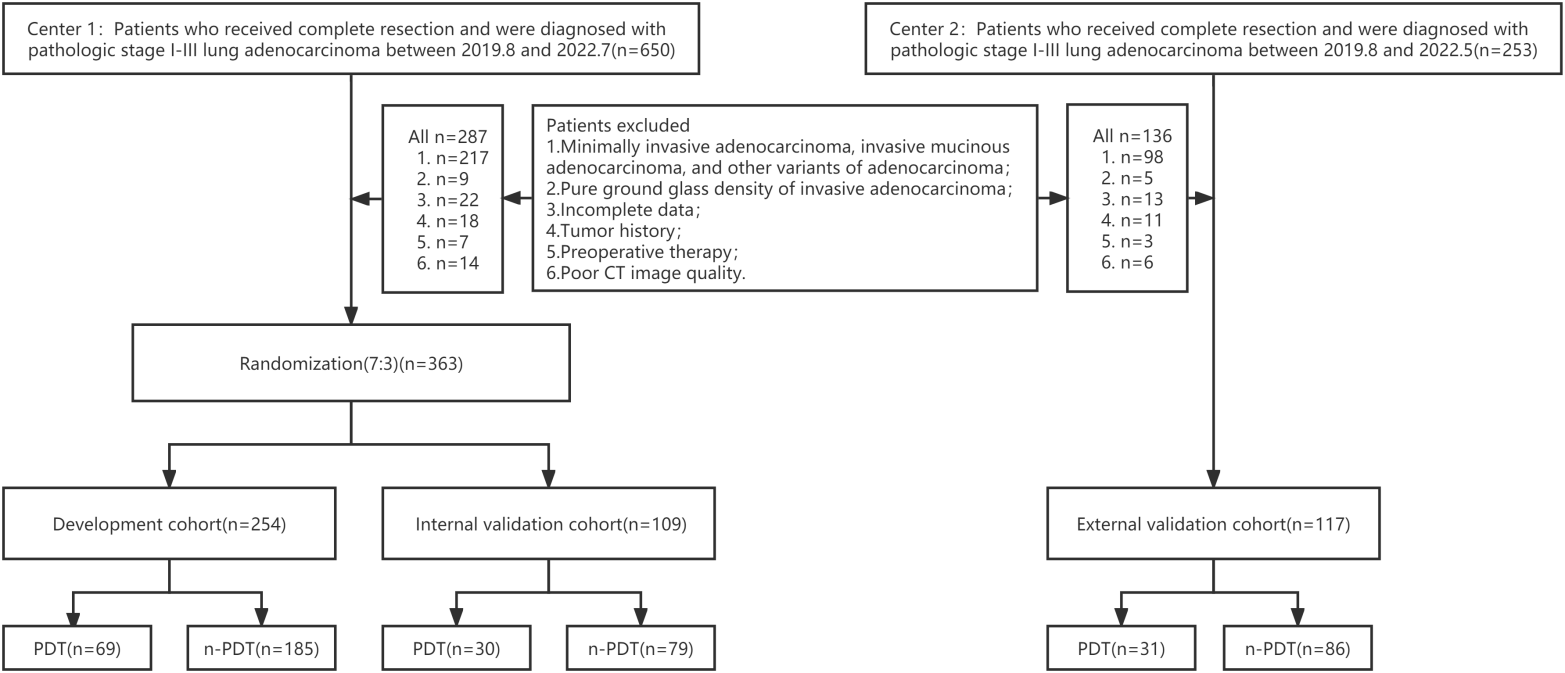
Flowchart of patient selection.

This retrospective, double-center study waived patients’ informed consent, authorized by the hospital’s Ethics Committee. Data were collected from two medical centers in China. Patients with stage I-III IPA manifesting as subsolid or solid lesions who underwent thoracic surgical resection were encompassed (specific exclusion criteria are shown in **Supplementary A1**). Eventually, 480 eligible patients (male 188, female 292) with IPA were included, aged 19-83 (mean age 63.4±9.9 years). 363 patients (142 male; 63.5 ± 9.8 years) from Center 1 (August 2019 to July 2022) were randomly divided into development cohort (n = 254; 106 male; 63.8 ± 9.6 years) and internal testing cohort (n = 109; 73 male; 62.2 ± 10.0 years) in a 7:3 ratio. And patients (n = 117; 71 male; 63.3 ± 10.4 years) from Center 2 (August 2019 to May 2022) were adopted as the independent external testing cohort. The study cohort flow diagram is shown in **Figure 1**. Histopathological evaluation, CT scan (**Supplementary Table 1**), and CT semantic features incorporated are exhibited in **Supplementary A2**.

### 2.2 Tumoral and peritumoral area segmentation

**Figure 2:**
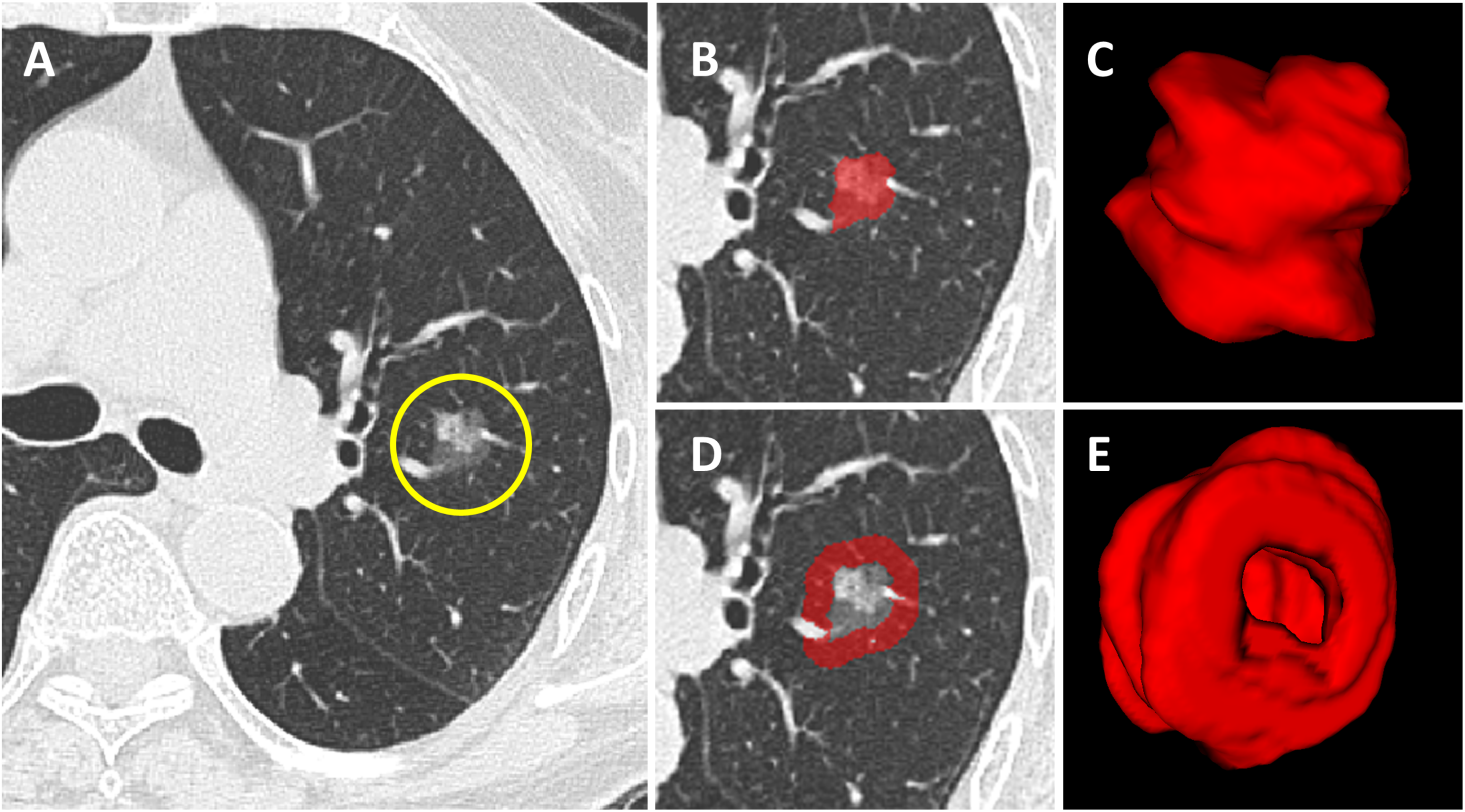
Tumoral and peritumoral area segmentation. **(A)** Computerized tomography (CT) image of a subsolid lesion pathologically confirmed as invasive lung adenocarcinoma. **(B)** The region of interest of the tumor is manually outlined layer by layer along the inner edge of the tumor. **(C)** The constructed 3D model of the intratumoral volume in ITK-SNAP. **(D)** The image showing the peritumoral region based on auto-dilating segmentation program, large blood vessels around the tumor are all excluded. **(E)** The constructed 3D model of the peritumoral volume in ITK-SNAP.

First, all lung CT DICOM images were imported into the open-source software ITK-SNAP (version 4.8; www.itksnap.org). Radiologists (Y.Z.B and D.H) with 10 years of experience in pulmonary imaging diagnosis from the two centers manually outlined the region of interest (ROI) layer by layer along the inner edge of the tumor until the whole tumor was covered. The results were fused and saved into 3D images, defined as intratumoral volume (ITV). The process above was scrutinized by an experienced chief radiologist (Z.F.H) with 20 years of experience in pulmonary imaging diagnosis. Any disagreement was settled through discussion. Large vessels, bronchi, cavities and spiculations were excluded from the ROI. None of the 3 radiologists was informed of the patient’s clinicopathological information. Subsequently, an automatic segmentation program(**Supplementary A3**) was applied to expand 5 mm outward to form the peri-ROI, defined as peritumoral volume (PTV). Large vessels and extrapleural normal tissue were ruled out from the peri-ROI (**Figure 2**). In our experiments, we utilized the Python programming language for data preprocessing and model implementation. All code was developed and tested under Python 3.7.7.

### 2.3 Consistency and repeatability evaluation

Three months later, two radiologists (Y.Z.B and D.H) randomly selected images of 40 patients from the development cohort. They segmented tumors and extracted features using an identical method but were unaware of each other’s segmentation process. The accuracy of 40 volumns from two segmentors was evaluated by calculating the Dice similarity coefficient. The intraclass correlation coefficient (ICC) was determined to appraise the reproducibility of tumor segmentation and feature extraction. ICC > 0.8 indicated excellent consistency and was included in the following research.

### 2.4 Radiomics features extraction, selection, and model construction

Radiomics features were retrieved using open-source PyRadiomics (V2.1.0). Image pre-processing, data-balancing, feature extraction (**Supplementary Table 2**), and screening are shown in **Supplementary A4**. Intratumoral radiomics signature (I-RS) and peritumoral radiomics signature (P-RS) models were constructed by retrieving and screening optimal features from ITV and PTV, respectively. Eventually, I-RS and P-RS were integrated to establish a combined radiomics model (IP-RS) via stepwise multivariate logistic regression based on the Akaike information criteria (AIC). AIC aims to minimize the number of parameters in a model while ensuring good fit, allowing the model to better adapt to new data and enhance generalization capability. In this study, we will select the model with the lowest AIC value as the approximate optimal model under the identical conditions.

### 2.5 Clinical CT semantic features screening and nomogram plotting

Univariate analysis was executed to probe the linkage between clinical CT semantic features and tumor differentiation grade. Variables with statistical differences (p < 0.05) were incorporated into the multivariate logistic regression to construct the clinical CT semantic model (C-C). Selected clinical CT semantic features were integrated with the combined radiomics model to plot a nomogram.

### 2.6 Statistical analysis

Statistical analysis were performed with R4.1.2 software. A t-test and Pearson*χ* ^2^ test or Fisher’s exact test were conducted to inspect the significance of clinical and CT semantic features. Receiver operator characteristic (ROC) curves were drawn. The area under the curve (AUC) and relevant indicators (accuracy, specificity, sensitivity, positive predictive value, and negative predictive value) were determined to comprehensively evaluate the predictive performance of each model. The DeLong test was carried out to statistically compare the differences in AUC between the nomogram and other models. AIC), Bayesian information criterion (BIC), and Root mean square error (RMSE) were adopted to assess the model goodness of fit and prediction performance. The Hosmer-Lemeshow test evaluated the calibration capability of the nomogram, which was visualized using the calibration curve. Moreover, we performed a decision curve analysis (DCA) to clarify the practicability and clinical usefulness. Two-sided p < 0.05 was referred to as statistical significance.

## 3 Results

### 3.1 Baseline characteristics and clinical CT semantic model construction

The baseline characteristics of the patients are shown in **Table 1**. In the development cohort, univariate analysis illustrated statistical differences (p < 0.05) in age, long diameter, pleural retraction, shape, density, spiculation, lobulation, bronchial obstruction, etc. Multivariate logistic regression analysis denoted that long diameter (OR: 1.126, 95% CI 1.073-1.183, p < 0.001, cut-off=19.5mm) and density (OR: 36.695, 95% CI 11.093-121.383, p < 0.001) were independent predictors of PDT (**Table 2**). A C-C model was generated using the two predictors. AUC values were 0.789(95% CI, 0.757-0.822) and 0.829(95% CI, 0.801-0.855) in internal testing cohort and external testing cohort, respectively. Additional detailed results are shown in **Table 3**.

**Table 1:** Baseline characteristics in three cohorts.

**Variable**	**Development Cohort** **(N=254)**		***p* value**	**Internal Testing Cohort (N=109)**		***p* value**	**External testing Cohort (N=117)**		***p* value**
	**PDT** **(N=69)**	**n-PDT** **(N=185)**		**PDT** **(N=30)**	**n-PDT** **(N=79)**		**PDT** **(N=31)**	**n-PDT** **(N=86)**	
Age(year)	61.8±9.4	64.5±9.6	0.046*	64.2±10.9	62.2±10.0	0.366	65.1±9.8	62.7±10.5	0.273
Gender									
Male	33 (31.1)	73 (39.5)	0.229	14 (46.7)	59 (74.7)	0.005*	14 (45.2)	57 (66.3)	0.039*
Female	36 (52.2)	112 (60.5)		16 (53.3)	20 (25.3)		17 (54.8)	29 (33.7)		
Pulmonary emphysema									
No	60 (87.0)	13 (7.0)	0.129	25 (83.3)	77 (97.5)	0.024*	24 (77.4)	82 (95.3)	0.010*
Yes	9 (13.0)	172(93.0)		5 (16.7)	2 (2.5)		7 (22.6)	4 (4.7)		
Smoking history									
No	45 (65.2)	137 (74.1)	0.165	21 (70.0)	69 (87.3)	0.033*	17 (54.8)	66 (76.7)	0.021*
Yes	24 (34.8)	48 (25.9)		9 (30.0)	10 (12.3)		14 (45.2)	20 (23.3)		
Location									
Left upper lobe	21 (30.4)	54 (29.2)	0.732	9 (30.0)	22 (27.8)	0.917	6 (19.4)	23 (26.7)	0.945
Left lower lobe	11 (15.9)	27 (14.6)		4 (13.3)	10 (12.7)		6 (19.4)	14 (16.3)		
Right upper lobe	22 (31.9)	71 (38.4)		10 (33.3)	21 (26.6)		11 (35.5)	28 (32.6)		
Right middle lobe	6 (8.7)	18 (9.7)		3 (10)	10 (12.7)		3 (9.7)	7 (8.1)		
Right lower lobe	9 (13.0)	15 (8.1)		4 (13.3)	16 (20.3)		5 (16.1)	14 (16.3)		
Density									
Part solid nodule	43 (62.3)	180 (97.3)	< 0.001**	15 (50)	76 (96.2)	< 0.001**	14 (45.2)	75 (87.2)	< 0.001**
Solid nodule	26 (37.7)	5 (2.7)		15 (50)	3 (3.8)		17 (54.8)	11 (12.8)		
Long diameter (mm)	23.2±9.2	16.0±6.2	< 0.001**	22.0±8.6	16.5±6.3	0.039*	22.6±9.4	16.1±6.0	< 0.001**
Pleural retraction									
No	15 (21.7)	85 (45.9)	< 0.001**	11 (36.7)	37 (46.8)	0.340	9 (29.0)	49 (57.0)	0.008*
Yes	54 (78.3)	100 (54.1)		19 (63.3)	42 (53.2)		22 (71.0)	37 (43.0)		
Shape									
Round or oval	8 (11.6)	44 (23.8)	0.032*	4 (13.3)	21 (26.6)	0.142	2 (6.5)	30 (34.9)	0.005*
Irregular	61 (88.4)	141 (76.2)		26 (86.7)	58 (73.4)		29 (93.5)	56 (65.1)		
Vacuole sign									
No	43 (62.3)	115 (62.2)	0.982	16 (53.3)	59 (74.7)	0.032*	29 (93.5)	70 (81.4)	0.188
Yes	26 (37.7)	70 (37.8)		14 (46.7)	20 (25.3)		2 (6.5)	16 (18.6)		
Spicule sign									
No	8 (11.6)	84 (45.4)	< 0.001**	2 (6.7)	39 (49.4)	< 0.001**	11 (35.5)	43 (50.0)	0.165
Yes	61 (88.4)	101 (54.6)		28 (93.3)	40 (50.6)		20 (64.5)	43 (50.0)		
Lobulation sign									
No	10 (14.5)	56 (30.3)	0.011*	3 (10.0)	37 (46.8)	0.001**	1 (3.2)	53 (61.6)	< 0.001**
Yes	59 (85.5)	129 (69.7)		27 (90.0)	42 (53.2)		30 (96.8)	33 (38.4)		
Bronchial obstruction									
No	19 (27.5)	98 (53.0)	< 0.001**	3 (10.0)	49 (62.0)	< 0.001**	9 (29.0)	47 (54.7)	0.014*
Yes	50 (72.5)	87 (47.0)		27 (90.0)	30 (38.0)		22 (71.0)	39 (45.3)		
Air bronchial sign									
No	49 (71.0)	119 (64.3)	0.316	25 (83.3)	51 (64.6)	0.057	21 (67.7)	43 (50.0)	0.089
Yes	20 (29.0)	66 (35.7)		5 (16.7)	28 (35.4)		10 (32.3)	43 (50.0)		

Data of age and long diameter are represented as mean ± standard deviation; other data are number of patients, with percentage in parentheses; PDT, Poorly Differentiated Tumor; n-PDT, non-Poorly Differentiated Tumor; *, Significant at p<0.05; **, Significant at p<0.005.

**Table 2:** Multivariate Logistic regression analysis results.

Variable	B	SE	Wald	p value	OR	95%CI
Long Diameter	0.119	0.025	22.714	< 0.001**	1.126	1.073~1.183
Density	3.603	0.610	34.837	< 0.001**	36.695	11.093~121.383
Constant	-4.885	0.645	57.421	< 0.001**	0.008	–

B, beta; SE, standard error; OR, odds ratio; CI, confidence interval; **, Significant at p<0.005.

**Table 3:** Prediction performance of the five models in the Internal Testing Cohor and External Testing Cohort.

	Model	AUC (95%CI)	Accuracy	Specificity	Sensitivity	PPV	NPV
**Internal Testing Cohort**	C-C	0.789(0.757-0.822)	0.771	0.875	0.568	0.700	0.797
	I-RS	0.838(0.806-0.867)	0.807	0.903	0.622	0.767	0.823
	P-RS	0.858(0.828-0.886)	0.826	0.917	0.649	0.800	0.835
	IP-RS	0.906(0.884-0.926)	0.826	0.955	0.623	0.900	0.797
	Nomogram	0.921(0.899-0.939)	0.853	0.943	0.684	0.867	0.848
**External Testing Cohort**	C-C	0.829(0.801-0.855)	0.795	0.878	0.600	0.677	0.837
	I-RS	0.893(0.871-0.913)	0.855	0.906	0.719	0.742	0.895
	P-RS	0.850(0.821-0.876)	0.803	0.944	0.587	0.871	0.779
	IP-RS	0.886(0.864-0.907)	0.821	0.922	0.625	0.806	0.826
	Nomogram	0.887(0.866-0.909)	0.846	0.914	0.686	0.774	0.872

PPV, Positive predictive values; NPV, Negative predictive values; AUC, Area under the receiver operating characteristics curve; CI, Confidence interval. I-RS, radiomics signature of the intratumoral region; P-RS, radiomics signature of the peritumoral region; IP-RS, combined radiomics signature of the intratumoral region and peritumoral region; C-C, clinical CT semantic signature.

### 3.2 Radiomics features extraction and consistency analysis

Here 1045 features were extracted from ITV and PTV, respectively. Dice similarity coefficient of 40 volumns from two segmentors was 0.796 ± 0.071. The consistency analysis manifested 940 (89.9%) features in ITV and 964 (92.2%) features in PTV with ICC> 0.8. The features above were used for further analysis.

### 3.3 Radiomics model construction and evaluation

**Figure 3:**
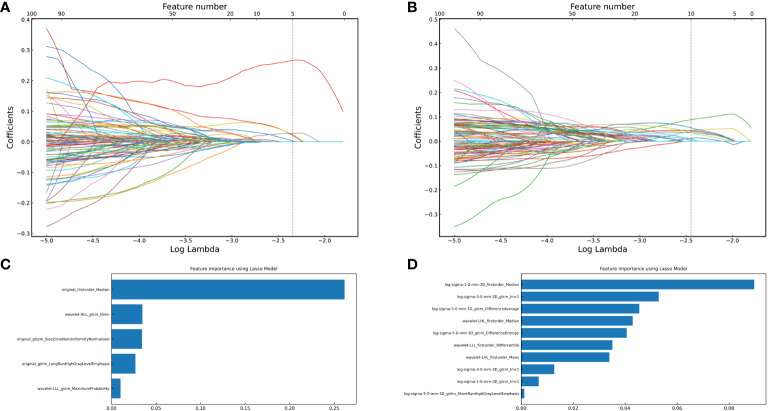
The optimal features were obtained using the Least absolute shrinkage and selection operator regression method with 5-fold cross-validation. **(A)** vertical dashed line indicates the best model fitted when lambda=0.085, and five optimal features of the intratumoral region; **(B)** vertical dashed line indicates the best model fitted when lambda=0.068, and ten optimal features of the peritumoral region; **(C)** Five optimal features of intratumoral radiomics model and corresponding weight coefficients (the weight coefficients value of the X-axis represents the importance of the featurs); **(D)** Ten optimal features of peritumoral radiomics model and corresponding weight coefficients.

**Figure 4:**
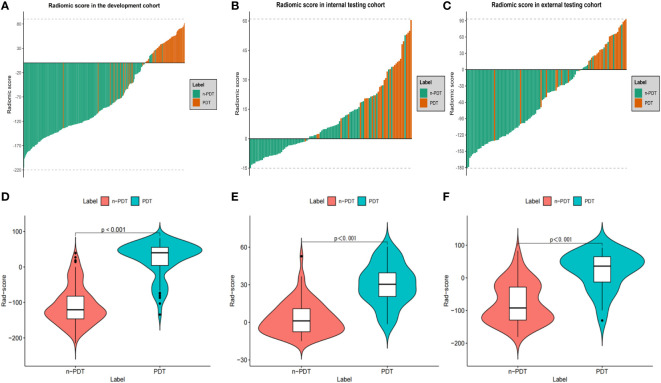
Waterfall plots and violin plots of the Rad-score in three cohorts. Waterfall plots of the Rad-score distribution for patients in development cohort **(A)**, internal testing cohort **(B)** and external testing cohort **(C)**. Violin plots of the Rad-score between PDT and n-PDT patients in development cohort **(D)**, internal testing cohort **(E)** and external testing cohort **(F)**. P-values between with PDT and n-PDT are placed in top of each image **(D–F)**.

After removing features with poor repeatability, the Spearman correlation coefficient was calculated, and redundant features with a correlation > 0.8 were removed. Features were ranked by minimum redundancy maximum relevance (mRMR), and the top 100 non-redundant features were selected. The optimal features were determined using the least absolute shrinkage and selection operator (Lasso) method with five-fold cross-validation. Finally, the five optimal features (original_firstorder_Median, wavelet-HLL_glcm_Idmn, original_glszm_SizeZoneNonUniformityNormalized, original_glrlm_LongRunHighGrayLevelEmphasis, wavelet-LLL_glcm_MaximumProbability) were extracted from the ITV region. Ten optimal features(log-sigma-1-0-mm-3D_firstorder_Median, log-sigma-5-0-mm-3D_glcm_Imc1, log-sigma-5-0-mm-3D_glcm_DifferenceAverage, wavelet-LHL_firstorder_Median, log-sigma-5-0-mm-3D_glcm_DifferenceEntropy, wavelet-LLL_firstorder_90Percentile, wavelet-LHL_firstorder_Mean, log-sigma-3-0-mm-3D_glcm_Imc1, log-sigma-1-0-mm-3D_glcm_Imc1, log-sigma-5-0-mm-3D_glrlm_ShortRunHighGrayLevelEmphasis) were extracted from PTV region. I-RS and P-RS models were constructed based on the best features screened by ITV and PTV (**Figure 3**). A combined radiomics model (IP-RS) was constructed based on intratumoral and peritumoral signatures. The correlation matrix of features in IP-RS model were exhibited in **Supplementary Figure 1**. The radiomics score (Rad-score) was calculated according to the weight coefficient of the model. All model feature weighting coefficients, intraclass correlation coefficient and the formula for calculating the radiomics score were shown in **Supplementary Table 3**. Substantial differences were observed in Rad-score between PDT and n-PDT groups via waterfall and violin plots (p < 0.01) (**Figure 4**). The AUCs of I-RS, P-RS, and IP-RS models in internal testing cohort were 0.838 (95% CI, 0.806-0.867), 0.858 (95% CI, 0.828-0.886), 0.906 (95% CI, 0.884-0.926), respectively. The AUC value of the IP-RS model was higher than that of the I-RS model. The DeLong test showed that there was no statistically significant difference between the AUCs of the IP-RS model and the I-RS model (0.906 vs 0.838, p=0.165). AUCs in the external testing cohort were 0.893 (95% CI, 0.871-0.913), 0.850 (95% CI, 0.821-0.876), and 0.886 (95% CI, 0.864-0.907), correspondingly (**Table 3**). The ROC curves were exhibited in **Supplementary Figure 2**.

### 3.4 Nomogram construction, internal testing, and external testing

**Figure 5:**
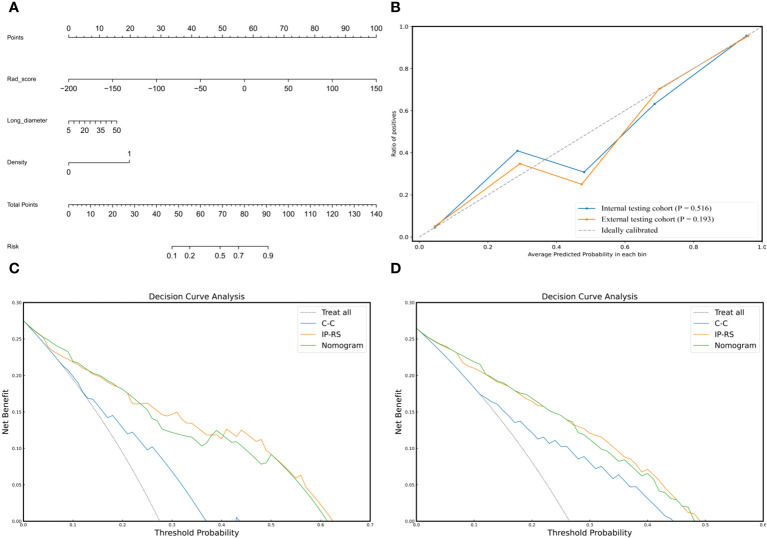
Composition, calibration and clinical applicability of the nomogram. **(A)** The individualized nomogram was generated by merging Rad-score and two traditional CT semantic features. **(B)** Calibration curves indicated good calibration ability of the nomogram in internal testing cohort and external testing cohort. Hosmer-Lemeshow test was applied in two cohorts (p= 0.516 in internal testing cohort; p=0.193 in external testing cohort). **(C, D)** DCAs of the prediction models in internal testing cohort **(C)** and external testing cohort **(D)**. The Y-axis represents the net benefit, and the x-axis shows the threshold probability. The DCA indicate that the nomogram and IP-RS model provide higher net benefits than C-C model in predicting the poorly differentiated invasive adenocarcinoma in the majority of areas. IP-RS, combined radiomics signatures of the intratumoral region and peritumoral region; C-C, clinical CT semantic signature; DCA, decision curve analysis.

**Figure 6:**
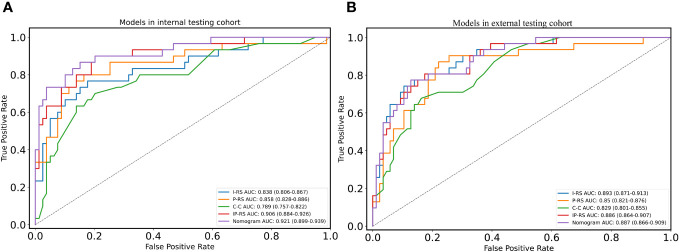
ROC curves of five models in two cohorts. **(A)** ROC curves of five models in internal testing cohort; **(B)** ROC curves of five models in external testing cohort. ROC, receiver operating characteristic; I-RS, radiomics signature of the intratumoral region; P-RS, radiomics signature of the peritumoral region; IP-RS, combined radiomics signature of the intratumoral region and peritumoral region; C-C, clinical CT semantic signature.

To construct a clinically applicable predictive model, the two independent predictors of the C-C model were merged with the combined radiomics model (IP-RS) in internal testing cohort to construct a nomogram (**Figure 5A**). The AUCs of the nomogram in internal testing cohort and external testing cohort were 0.921 (95% CI, 0.899-0.939) and 0.887 (95% CI, 0.866-0.909), respectively. ROC curves of the nomogram and other models are displayed in **Figure 6**. Specific performance indicators of each model are shown in **Table 3**. The Delong test showed that the AUC of the nomogram was significantly higher than that of the C-C model in both the internal testing cohort(0.921 vs 0.789, p< 0.05) and external testing cohort(0.887 vs 0.829, p< 0.05). Although there was no statistically significant difference in AUC between the nomogram and I-RS(0.921 vs 0.838, p= 0.085), P-RS(0.921 vs 0.858, p= 0.182) and IP-RS(0.921 vs 0.906, p= 0.404) models in the internal testing cohort, the AIC and BIC values (**Table 4**) of the nomogram were the lowest, indicating that the nomogram bore the best goodness of fit. A smaller RMSE value denotes that the nomogram has the closest predictions to the actual scenarios, i.e., the model’s prediction is more accurate (**Table 4**). Therefore, combined with AUC, AIC, BIC, and RMSE, the nomogram showed the best fitting and prediction accuracy. The Hosmer-Lemeshow test and calibration curve indicated good calibration ability of the nomogram (**Figure 5B**). The DCA (**Figures 5C, D**) showed that the nomogram and radiomics models had higher clinical nets benefit than the C-C model.

**Table 4:** Performance and goodness-of-fit evaluation of models.

models	AIC	BIC	RMSE	DeLong Test (compared with Nomogram)
C-C	-122.984	-117.601	0.559	0.028*
I-RS	-170.433	-165.051	0.449	0.085
P-RS	-156.499	-151.116	0.479	0.182
IP-RS	-179.641	-174.236	0.432	0.404
Nomogram	-180.822	-175.439	0.421	/

AIC, Akaike information criterion; BIC, Bayesian information criterion; RMSE, Root mean square error; *, Significant at p<0.05.

## 4 Discussion

In this two-center study, we trained a nomogram using intratumoral and peritumoral radiomics features combined with clinical CT semantic features. This nomogram obtained favorable outcomes in both the internal testing cohort and the independent external testing cohort, reflecting the model’s excellent predictive capacity for PDT before surgery. The nomogram presented the highest AUC and the lowest AIC, BIC, and RMSE in internal testing cohort. This substantiated that the combined model exhibited higher predictive power and superior goodness of fit versus the radiomics or clinical semantic models alone. The high net profit of the DCA also underpinned the clinical application value of the nomogram.

Univariate and multivariate analyses of semantic features of clinical CT revealed that long diameter and density were PDT’s final independent predictors. This aligns with the general understanding that poorer tumor differentiation represents stronger invasiveness, faster growth, and thus larger tumor size, consistent with the results of many studies (22, 23). Moreover, this underlies the guideline’s stratification on lung cancer patients’ prognoses (27). Several confirmatory studies of the novel IASLC grading system found that pure solid tumors were mostly PDT (12, 28, 29). Fujikawa et al. (12) reported that density was still an effective predictor of PDT after excluding confounders and bias, which agrees with our findings. Larger solid tumors were defined as PDT, simple common sense. Accurate screening of PDT is challenging since doctors often encounter small solid or large subsolid tumors in clinical scenarios, underpinned by an AUC of 0.789 of the C-C model in internal testing cohort. Therefore, we focused on exploring the potential of radiomics to generate better and more generalizable models by combining clinical information.

The six radiomics features in the final model comprised two first-order statistical features and four texture features, including two Gray Level Co-occurrence Matrixes (GLCM), one Gray Level Run Length Matrix (GLRLM), and one Gray Level Size Zone Matrix (GLSZM). First-order statistical features represent the voxel intensity distribution within the image area defined by the mask, which is highly correlated with our model and may denote more significant tumoral heterogeneity. Yang et al. illustrated that first-order statistical features were the most substantial predictors of tumor serosal invasion (30). Another study also revealed that first-order statistical features could predict the differentiation degree of IPA (22). GLCM is the nomogram’s predominant texture, representing the heterogeneity between images by calculating the speed and amplitude of the variations of two pixels in different intervals and directions. PDT is more invasive and contains more hypoxia-induced necrosis, leading to more significant tumor heterogeneity quantified by CT texture analysis (31). Li et al. (23) reported that texture features like GLCM performed well in predicting the differentiation degree of IPA, congruous with our results.

There are few related radiomics studies since the IASLC grading system has merely been applied in clinical practice for a short time. Li et al. (23) established radiomics and quantitative semantic models using low-dose computed tomography (LDCT) to predict PDT, with AUCs of 0.921 and 0.923 in the training set, respectively. Yang et al. (22) devised a nomogram model based on CT-based radiomics combined with clinical and radiological features to predict novel IASLC classifications with an AUC of 0.915 in the development cohort and 0.838 in testing cohort. Significant AUC reduction indicates an unstable model with potential overfitting. Nevertheless, their study has an apparent shortcoming: omitting the predictive effect of peritumoral information or external testing of independent data cohorts. This raises questions about the generalization ability of their models. Since Lambin et al. proposed the concept of radiomics (32), model generalization has been a focus for research improvement. Recently, multi-center data and the introduction of independent external testing cohorts have been emphasized, which can fix models’ poor generalization capability to some extent (33). Our nomogram in the internal testing cohort exhibited excellent performance(AUC = 0.921), similar to the results reported in the two aforementioned articles. The nomogram in the independent external testing cohort still achieved an AUC of 0.887, indicating that our model performs well and possesses good generalization ability. Additionally, the DeLong test revealed that the predictive performance of nomogram were significantly better than the C-C model in internal testing cohort and external testing cohort (both p < 0.05). Thus, the nomogram can assist thoracic surgeons to better judge the differentiation degree of IPA before operation, compared to traditional clinical semantic model.

In recent years, increasing attention has been directed to the peritumoral microenvironment (18, 34). Shimada et al. (17) found that peritumoral lymphovascular invasion could better reflect hematogenous metastasis as an essential predictor of prognosis and distant metastasis. Beig et al. (23)demonstrated that massive tumor-infiltrating lymphocytes and tumor-associated macrophages resided around IPA, predominantly showing smooth texture on CT images. Additionally, he pointed out that texture features within 5 mm around the tumor performed best in predicting benign and malignant solid pulmonary nodules. Our definition of peritumoral area is mainly based on their results, consistent withWu et al.’s definition (35). In internal testing cohort, we integrated peritumoral radiomic features into the I-RS model to construct the IP-RS model, which increased the AUC value from 0.838 to 0.906. However, DeLong’s test showed no statistically significant difference between the two (p = 0.165). We speculate that there may be several reasons for these findings: Firstly, the heterogeneity of peritumoral radiomic features between different differentiations of IPAs may not be as distinct as that observed between benign and malignant tumors. Secondly, our definition of a peritumoral range of 5mm may be insufficient, as varying peritumoral ranges could potentially influence the model’s performance (19, 36). Lastly, our peritumoral dilation is based on 2D, and it may lead to the loss of valuable information compared to 3D dilation. In addition, we noted that the AUCs for the IP-RS model in the internal testing cohort and external testing cohort were 0.858 and 0.850, respectively, with the values being nearly identical. Khorrami et al. (37) showed that peritumoral radiomic features were less affected by the scanner parameters compared to intratumoral features. They suggested that peritumoral features were more stable than intratumoral features in differentiating IPA from granuloma. Therefore, the additive effect of peritumoral radiomics on intratumoral radiomics may be limited, but peritumoral radiomics has the potential to enhance the stability of the combined model. The nomogram incorporating peritumoral radiomic features did not show a significant decrease in AUC in an independent external testing cohort. This indirectly reflects this aspect, which greatly contributes to improving the model’s generalization capability.

There are several shortcomings in this study. To begin with, as a retrospective study, this study bears inevitable potential selection bias. Prospective, high-quality, multi-center studies are required to corroborate this nomogram and promote clinical application. Second, CT image acquisition was not uniform for patients from the two centers. We lacked the application of methods like ComBat to harmonize the dual-center data. We will harmonize the multi-center data in subsequent research, contributing to the reliability and reproducibility of experimental results. However, all images are pre-processed, e.g., resampling and normalization before feature extraction. The nomogram worked well in independent external testing cohort, indicating that our model has good generalization ability. Last, the peritumoral range was only defined as 5 mm, which could be one reason why peritumoral radiomics didn’t show significantly additive effect to intratumoral radiomics. In the future, we will delve into varying ranges to characterize peritumoral data to better assess their role in predicting the differentiation degree of IPA.

## 5 Conclusions

We substantiated that the nomogram based on intratumoral and peritumoral radiomics features and clinical CT semantic features could effectively determine the differentiation degree of IPA manifesting as subsolid or solid lesions. The nomogram can serve as a non-invasive, repeatable personalized tool for preoperative assessment of IPA differentiation degree.

## Data availability statement

The original contributions presented in the study are included in the article/**Supplementary Material**. Further inquiries can be directed to the corresponding authors.

## Ethics statement

The studies involving humans were approved by Dongyang Hospital ethics review board. The studies were conducted in accordance with the local legislation and institutional requirements. Written informed consent for participation was not required from the participants or the participants’ legal guardians/next of kin in accordance with the national legislation and institutional requirements. Written informed consent was obtained from the individual(s) for the publication of any potentially identifiable images or data included in this article.

## Author contributions

ZY: Conceptualization, Data curation, Funding acquisition, Investigation, Methodology, Resources, Visualization, Writing – original draft. HD: Conceptualization, Data curation, Investigation, Software, Validation, Writing – review & editing. CF: Data curation, Formal Analysis, Methodology, Project administration, Resources, Writing – review & editing. ZZ: Data curation, Formal Analysis, Methodology, Resources, Software, Writing – review & editing. YH: Data curation, Investigation, Writing – review & editing. KS: Conceptualization, Investigation, Writing – review & editing. CM: Data curation, Formal Analysis, Writing – review & editing. XC: Conceptualization, Data curation, Writing – review & editing. JX: Data curation, Investigation, Writing – review & editing. ZP: Data curation, Investigation, Writing – review & editing. MH: Funding acquisition, Project administration, Resources, Writing – review & editing. XZ: Data curation, Resources, Writing – review & editing. WZ: Formal Analysis, Resources, Supervision, Writing – review & editing. LL: Data curation, Formal Analysis, Writing – review & editing. WL: Funding acquisition, Project administration, Resources, Supervision, Visualization, Writing – review & editing. JS: Funding acquisition, Project administration, Resources, Software, Supervision, Visualization, Writing – review & editing. FZ: Funding acquisition, Investigation, Resources, Supervision, Visualization, Writing – review & editing, Conceptualization, Data curation, Formal Analysis.
