# Supplementary material for: A nomogram based on CT intratumoral and peritumoral radiomics features preoperatively predicts poorly differentiated invasive pulmonary adenocarcinoma manifesting as subsolid or solid lesions: a double-center study
Source: Front Oncol. 2024 Jan 19;14:1289555. doi: 10.3389/fonc.2024.1289555 (PMC10834705; doi:10.3389/fonc.2024.1289555)
Supplement: Supplementary file 1 [file DataSheet_1.docx]

**Supplementary file**

**Supplementary A1**: Exclusion criteria of I-III stage IPA patients.

**Supplementary A2**: Inclusion of patients' histopathological appraisal, CT scan, and CT semantic features.

**Supplementary A3**: automatic segmentation program.

**Supplementary A4**: Image preprocessing, data balancing, radiomics features extraction and screening.

**Supplementary Table 1**: The CT protocol of the two independent centers.

**Supplementary Table 2**: Different filter and radiomics features

**Supplementary Table 3**: All model feature weighting coefficients, Intraclass correlation coefficient and Rad-score calculation formula

**Supplementary Figure 1**: The correlation matrix of radiomics features

**Supplementary Figure 2**: ROC curves of radiomics and clinical CT semantic signatures in internal testing and external testing cohorts

**Supplementary A1: Exclusion criteria of I-III stage IPA patients.**

Specific exclusion criteria:

1. Patients with micro-invasive adenocarcinoma, pure ground-glass invasive adenocarcinoma, invasive mucinous adenocarcinoma, and other adenocarcinoma variants;

2. Patients with incomplete histopathological results or missing clinical data;

3. Patients underwent preoperative chemotherapy, radiotherapy, and biopsy;

4. Patients with prior lung tumors or who had a history of other malignancies;

5. Poor CT image quality that affected the qualitative assessment of tumors.

**Supplementary A2: Inclusion of patients' histopathological appraisal, CT scan, and CT semantic features.**

**Histopathological appraisal:** Surgically excised samples were fixed in formalin, embedded in paraffin, sectioned, and stained with hematoxylin and eosin (H&E). All available H&E-stained tumor sections were re-read and interpreted by two pathologists in accordance to the novel 2020 IASLC grading system. Since the proportion of histological subtypes in the specimen will be affected by the subjective experience of pathologists, divergences of pathological classification were settled through discussion. Three pathological subtypes: Grade1: lepidic predominant tumor with <20% of high-grade patterns (solid, micropapillary, or complex gland); Grade2: acinar or papillary predominant tumor with <20% of high-grade patterns; Grade3: any tumor with ≥20% of high-level patterns. Patients were classified into groups according to treatments and prognoses. Grade 3 had adverse prognoses and was defined as PDT. Grades 1 and 2 had good prognoses and were described as n-PDT.

**CT scan:** All CT images were based on high-resolution non-contrast scans. All patients were scanned with Philips Brilliance 64 CT and UIH uCT510 CT for preoperative baseline examination. Before scanning, all patients were given strict respiratory training in a head-first supine position. CT scans were obtained during end-inspiratory breath holds, covering the entire lungs from the apex to the base. Scanning parameters and image reconstruction were specified in Supplementary Table 1. The reconstruction kernel of CT images was determined using high spatial resolution kernel.

**CT semantic features:** Two senior radiologists (YZB and ZFH with ten years and twenty years of working experience, respectively) independently read the films without knowing the pathological results. They measured, evaluated, and recorded the CT semantic signs of the tumor. Any disagreements were resolved by discussion. Eligible CT semantic features: (1) tumor location; (2) long diameter (the diameter of the maximum cross-section area of the tumor on the lung window); (3) density (solid or part-solid); (4) shape (quasi-circular or irregular); (5) pleural retraction (absent or present); (6) spiculation sign (absent or present); (7) lobulation (absent or present); (8) vacuole sign (absent or present); (9) air bronchogram (absent or present); (10) bronchial obstruction (absent or present).

**Supplementary A3: automatic segmentation program.**

We developed an auto-dilating segmentation program using the Python programming language. All code was developed and tested under Python 3.7.7. This code implements a lung segmentation algorithm based on medical images. Firstly, the CT image array is preprocessed by binarizing the image through setting a threshold to distinguish air and anatomical structures. Subsequently, a connected threshold algorithm and morphological operations are employed to generate a binary mask representing the overall body shape, which is then inverted to highlight the lung region. Further refinement of the lung shape is achieved through binary morphological closing and connected threshold operations. Finally, the code generates the ultimate lung segmentation result by adjusting the weights of pre-generated lung segmentation labels, original segmentation results, and internal shape information. Finally, the code generates the ultimate dilated part of lung segmentation result by adjusting the weights of pre-generated lung segmentation labels, original segmentation results, and internal shape information. The key idea behind this code is to consider both global shape features and local details of the image to enhance the accuracy of lung segmentation.

**Supplementary A4: Image preprocessing, data balancing, radiomics features extraction and screening.**

**Image preprocessing:** To reduce the distinctions in image texture features between different machines, we first resampled all images to a spatial resolution of 1×1×1 mm^3^, and then Z-score normalized the data with data outside of mean ± 4SD eliminated. Eventually, preprocessed data were limited to 0-1 to exclude adverse effects caused by singular samples.

**Radiomics features extraction:** There were 6 kinds of radiomics features: 18 first-order statistical features, 14 shape features, 22 Gray Level Co-occurrence Matrix (GLCM), 16 Gray Level Size Zone Matrix (GLSZM), 16 Gray Level Run Length Matrix (GLRLM), and 14 Gray Level Dependence Matrix (GLDM)( Supplementary Table 2). Furthermore, Wavelet and Laplacian of Gaussian (LoG) filters are applied to the raw image to obtain the corresponding derivative images. Subsequently, radiomics features were acquired from the three types of images.

**Data balancing:** Since the n-PDT number exceeds PDTs in the development cohort (about 3:1), we adopted the synthetic minority oversampling technique (SMOTE) to balance a minority of samples in the training set, making the two training samples close to 1:1, reducing the potential impact of sample imbalance on the model. Dimensionality reduction and machine learning modeling were performed using balanced data. After modeling, all predictions were underpinned by actual internal testing and external testing cohort data.

**Radiomics features screening:** First, Spearman correlation analysis used features with ICC>0.8 to reduce the collinearity among features. Then, the minimum redundancy maximum correlation (mRMR) algorithm ranked the features. The top 100 most relevant features were acquired. Eventually, the optimal features were obtained using the Least absolute shrinkage and selection operator (Lasso) regression method with 5-fold cross-validation.

**Supplementary Table 1. The CT protocol of the two independent centers.**

| **Parameters** | **Center1** | **Center2** | |
| --- | --- | --- | --- |
| CT version | Philips Brilliance 64 | Philips Brilliance 64 | UIH uCT510 |
| CT tube voltage | 120 kVp | 120 kVp | 120 kVp |
| CT tube current | 150 mAs | 200 mAs | 140 mAs |
| CT rotation time | 0.6s | 0.7s | 0.5s |
| CT detector collimation | 64X0.625mm | 64X0.625mm | 16X0.625mm |
| Pitch | 0.64 | 0.64 | 0.9375 |
| Layer thickness | 5 mm | 2 mm | 3 mm |
| Reconstruction Algorithm | Hybrid iterative reconstruction (iDose4, level 6) | Hybrid iterative reconstruction (iDose4, level 6) | Dual-domain iterative reconstruction |
| Reconstruction thickness | 0.67mm | 1mm | 1mm |
| Reconstruction interval | 0.67mm | 1mm | 1mm |
| Image matrix | 1024*1024 | 1024*1024 | 1024*1024 |

**Supplementary Table 2. Different filter and radiomics features**

| **Filter(10)** | **Firstorder(18)** | **GLCM(22)** | **GLSZM(16)** |
| --- | --- | --- | --- |
| Original,  wavelet-LLH,  wavelet-LHL,  wavelet-LHH,  wavelet-HLL,  wavelet-HLH,  wavelet-HHL,  wavelet-HHH,  wavelet-LLL,  Laplacian of Gaussian (LoG). | 10Percentile,  90Percentile,  Energy,  Entropy,  InterquartileRange,  Kurtosis,  Maximum,  MeanAbsoluteDeviation,  Mean,  Median,  Minimum,  Range,  RobustMeanAbsoluteDeviation,  RootMeanSquared,  Skewness,  TotalEnergy,  Uniformity,  Variance. | Autocorrelation,  JointAverage,  ClusterProminence,  ClusterShade,  ClusterTendency,  Contrast,  Correlation,  DifferenceAverage,  DifferenceEntropy,  DifferenceVariance,  JointEnergy,  JointEntropy,  Imc1,  Imc2,  Idm,  Idmn,  Id,  Idn,  InverseVariance,  MaximumProbability,  SumEntropy,  SumSquares. | GrayLevelNonUniformity,  GrayLevelNonUniformityNormalized,  GrayLevelVariance,  HighGrayLevelZoneEmphasis,  LargeRegionEmphasis,  LargeRegionHighGrayLevelEmphasis,  LargeRegionLowGrayLevelEmphasis,  LowGrayLevelZoneEmphasis,  SizeZoneNonUniformity,  SizeZoneNonUniformityNormalized,  SmallRegionEmphasis,  SmallRegionHighGrayLevelEmphasis,  SmallRegionLowGrayLevelEmphasis,  ZoneEntropy,  ZonePercentage,  ZoneVariance. |
| **GLRLM(16)** | **GLDM(14)** | **SHAPE(14)** |  |
| GrayLevelNonUniformity,  GrayLevelNonUniformityNormalized,  GrayLevelVariance,  HighGrayLevelRunEmphasis,  LongRunEmphasis,  LongRunHighGrayLevelEmphasis,  LongRunLowGrayLevelEmphasis,  LowGrayLevelRunEmphasis,  RunEntropy,  RunLengthNonUniformity,  RunLengthNonUniformityNormalized,  RunPercentage,  RunVariance,  ShortRunEmphasis,  ShortRunHighGrayLevelEmphasis,  ShortRunLowGrayLevelEmphasis. | DependenceEntropy,  DependenceNonUniformity,  DependenceNonUniformityNormalized,  DependenceVariance,  GrayLevelNonUniformity,  GrayLevelVariance,  HighGrayLevelEmphasis,  LargeDependenceEmphasis,  LargeDependenceHighGrayLevelEmphasis,  LargeDependenceLowGrayLevelEmphasis,  LowGrayLevelEmphasis,  SmallDependenceEmphasis,  SmallDependenceHighGrayLevelEmphasis,  SmallDependenceLowGrayLevelEmphasis. | Elongation,  Flatness,  LeastAxisLength,  MajorAxisLength,  Maximum2DDiameterColumn,  Maximum2DDiameterRow,  Maximum2DDiameterSlice,  Maximum3DDiameter,  MeshVolume,  MinorAxisLength,  Sphericity,  SurfaceRegion,  SurfaceVolumeRatio,  VoxelVolume. |  |

**Supplementary Table 3. All model feature weighting coefficients, Intraclass correlation coefficient and Rad-score calculation formula**

| Model | Feature Name | Regression coefficient | Intraclass correlation coefficient |
| --- | --- | --- | --- |
| I-RS |  | β | ICC |
| I998 | wavelet-LLL_glcm_MaximumProbability | 0.0101 | 0.88 |
| I60 | original_glrlm_LongRunHighGrayLevelEmphasis | 0.0269 | 0.95 |
| I80 | original_glszm_SizeZoneNonUniformityNormalized | 0.0343 | 0.96 |
| I650 | wavelet-HLL_glcm_Idmn | 0.0347 | 0.94 |
| I24 | original_firstorder_Median | 0.2617 | 0.96 |
| P-RS |  | β | ICC |
| P327 | log-sigma-5-0-mm-3D_glrlm_ShortRunHighGrayLevelEmphasis | 0.0012 | 0.84 |
| P131 | log-sigma-1-0-mm-3D_glcm_Imc1 | 0.0067 | 0.97 |
| P217 | log-sigma-3-0-mm-3D_glcm_Imc1 | 0.0127 | 0.98 |
| P453 | wavelet-LHL_firstorder_Mean | 0.0339 | 0.95 |
| P962 | wavelet-LLL_firstorder_90Percentile | 0.0351 | 0.94 |
| P299 | log-sigma-5-0-mm-3D_glcm_DifferenceEntropy | 0.0406 | 0.99 |
| P454 | wavelet-LHL_firstorder_Median | 0.0429 | 0.94 |
| P298 | log-sigma-5-0-mm-3D_glcm_DifferenceAverage | 0.0455 | 0.98 |
| P303 | log-sigma-5-0-mm-3D_glcm_Imc1 | 0.0529 | 0.98 |
| P110 | log-sigma-1-0-mm-3D_firstorder_Median | 0.0897 | 0.99 |
| IP-RS |  | β | ICC |
| I998 | wavelet-LLL_glcm_MaximumProbability | 0.0006 | 0.88 |
| P453 | wavelet-LHL_firstorder_Mean | 0.0215 | 0.95 |
| I80 | original_glszm_SizeZoneNonUniformityNormalized | 0.0217 | 0.96 |
| I650 | wavelet-HLL_glcm_Idmn | 0.0238 | 0.94 |
| I60 | original_glrlm_LongRunHighGrayLevelEmphasis | 0.0252 | 0.95 |
| I24 | original_firstorder_Median | 0.2522 | 0.96 |
| C-C |  | β |  |
|  | Long diameter | 0.0777 |  |
|  | Density | 0.1690 |  |
| I-RS=I998*0.0101+I60*0.0269+I80*0.0343+I650*0.0347+I24*0.2617 | | |  |
| P-RS=P327*0.0012+P131*0.0067+P217*0.0127+P453*0.0339+P962*0.0351+P299*0.0406+P454*0.0429+P298*0.0455+P303*0.0529+P110*0.0897 | | |  |
| IP-RS=I998*0.0006+P453*0.0215+I80*0.0217+I650*0.0238+I60*0.0252+I24*0.2522 | | |  |
| C-C=Long diameter*0.777+Density*0.1690 | | |  |
| Abbreviations: I-RS, radiomics signature of the intratumoral region; P-RS, radiomics signature of the peritumoral region; IP-RS, combined radiomics signature of the intratumoral region and peritumoral region; C-C, clinical CT semantic signature.;β, regression coefficient; ICC, intraclass correlation coefficient. | | |  |

**Supplementary Figure 1. The correlation matrix of radiomics features**

Heat map of correlations among each feature in IP-RS model. The darker the color, the smaller the value, representing the lower the collinearity. IP-RS, combined radiomics signature of the intratumoral region and peritumoral region.

**Supplementary Figure 2. ROC curves of radiomics and clinical CT semantic signatures in internal testing and external testing cohorts.**

(A) ROC curves of C-C in internal testing and external testing cohorts; (B) ROC curves of I-RS in internal testing and external testing cohorts; (C) ROC curves of P-RS in internal testing and external testing cohorts; (D) ROC curves of the combined radiomics signature in internal testing and external testing cohorts. ROC, Receiver operating characteristic; I-RS, radiomics signature of the intratumoral region; P-RS, radiomics signature of the peritumoral region; IP-RS, combined radiomics signature of the intratumoral region and peritumoral region; C-C, clinical CT semantic signature.
