# Supplementary figures and images for: A nomogram based on CT intratumoral and peritumoral radiomics features preoperatively predicts poorly differentiated invasive pulmonary adenocarcinoma manifesting as subsolid or solid lesions: a double-center study
Source: Front Oncol. 2024 Jan 19;14:1289555. doi: 10.3389/fonc.2024.1289555 (PMC10834705; doi:10.3389/fonc.2024.1289555)

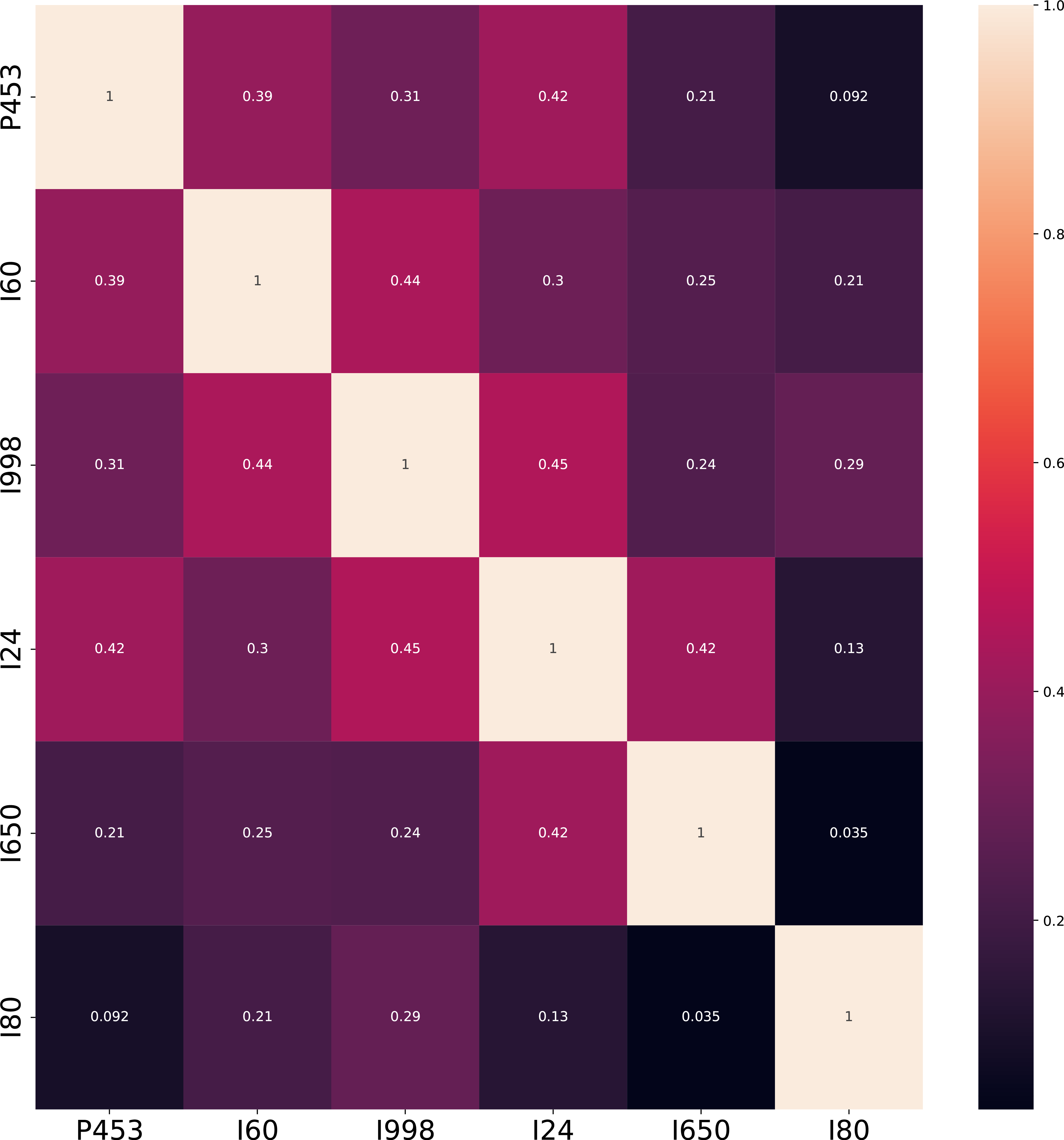

Supplement: Supplementary file 2 [file Image_1.tif]

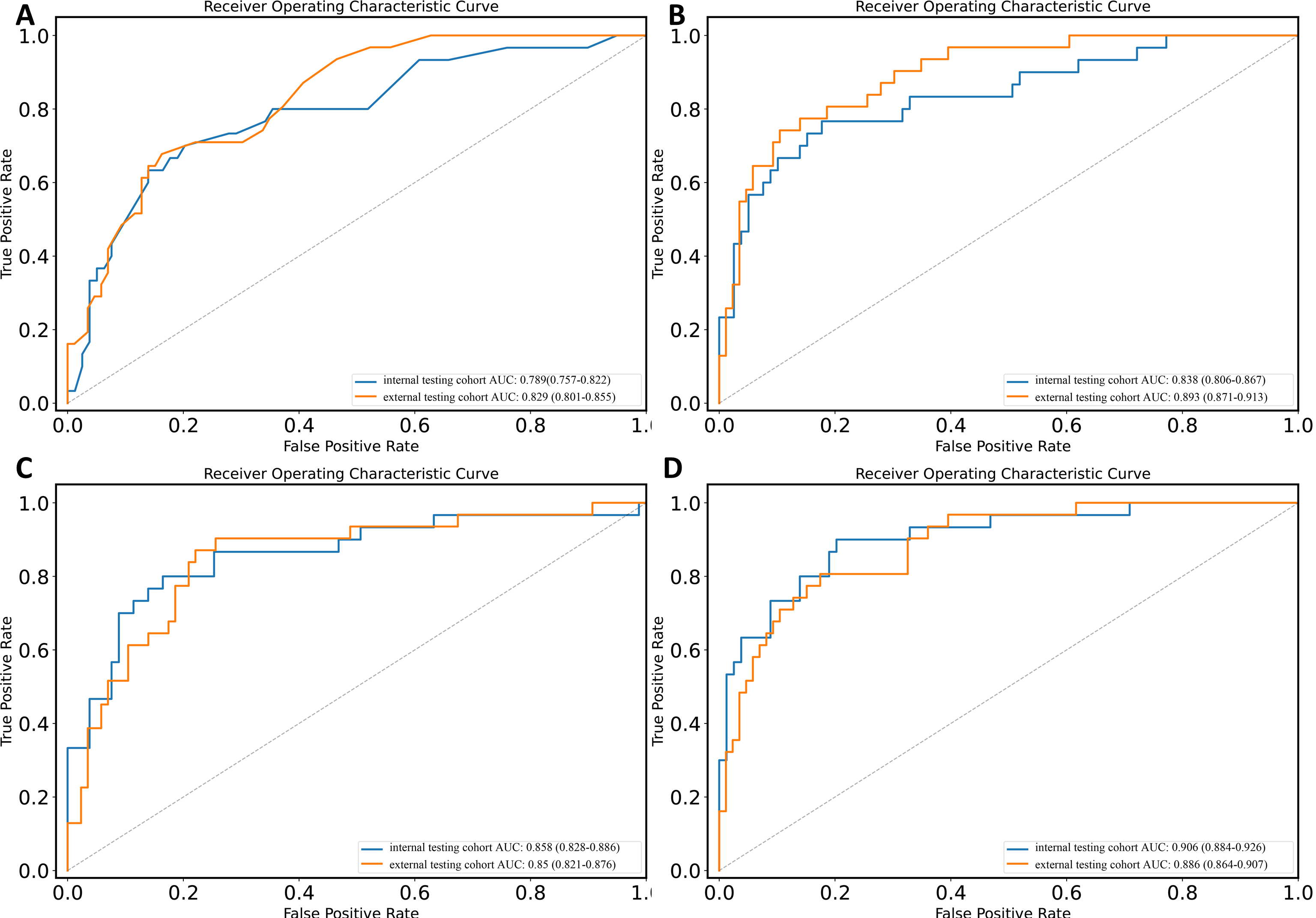

Supplement: Supplementary file 3 [file Image_2.tif]
